# Enhanced detection of gaze toward an object: Sociocognitive influences on visual search

**DOI:** 10.3758/s13423-020-01841-5

**Published:** 2020-11-10

**Authors:** Nayantara Ramamoorthy, Oliver Jamieson, Nahiyan Imaan, Kate Plaisted-Grant, Greg Davis

**Affiliations:** Department of Psychology, University of Cambridge, Cambridge, UK

**Keywords:** Visual search, Gaze perception, Gaze-object relations, Social processing

## Abstract

Another person’s gaze direction is a rich source of social information, especially eyes gazing toward prominent or relevant objects. To guide attention to these important stimuli, visual search mechanisms may incorporate sophisticated coding of eye-gaze and its spatial relationship to other objects. Alternatively, any guidance might reflect the action of simple perceptual ‘templates’ tuned to *visual* features of socially relevant objects, or intrinsic salience of direct-gazing eyes for human vision. Previous findings that direct gaze (toward oneself) is prioritised over averted gaze do not distinguish between these accounts. To resolve this issue, we compared search for eyes *gazing toward* a prominent object versus *gazing away*, finding more efficient search for eyes ‘gazing toward’ the object. This effect was most clearly seen in target-present trials when gaze was task-relevant. Visual search mechanisms appear to specify gazer-object relations, a computational building-block of theory of mind.

## Introduction

The human eye’s marked dark-iris, light-sclera morphology (Kobayashi & Kohshima, 1997) offers a salient and important social signal (e.g., Cañigueral & Hamilton, 2019; Senju & Johnson, 2009). Perception of another’s eye gaze activates a large-scale social-cognition network in the human brain (e.g. Carlin & Calder, 2013; McCrackin & Itier, 2019), and is considered a foundation for social development (e.g., Baron-Cohen, 1994; Baron-Cohen, 2005; Charman et al., 2000; Tomasello, Carpenter, Call, Behne, & Moll, 2005). Given their crucial importance, one would expect visual attention to be drawn toward eyes, when they are present. Not all eyes need be of equal priority, however. Those gazing toward us (‘direct gaze’) or at other relevant objects and events may be particularly informative and so prioritised over ‘other gaze’ during search.

Previous work on this topic has largely compared *direct* gaze (directed to the observer) with *averted gaze* (directed elsewhere). Direct gaze, assumed to be of greater importance, has been reported to engage attention more effectively (Senju & Hasegawa, 2005; Ueda, Takahashi, & Watanabe, 2014), even when task-irrelevant (Böckler, van der Wel, & Welsh, 2014; Doi & Shinohara, 2013; Yokoyama, Sakai, Noguchi, & Kita, 2014). Moreover, in visual search, direct-gazing eyes are typically detected more rapidly among averted-gazing distracters than vice versa — a ‘stare-in-the-crowd’ effect (SITCE; Conty, Tijus, Hugueville, Coelho, & George, 2006; Ramamoorthy, Plaisted-Grant, & Davis, 2019; Senju, Hasegawa, & Tojo, 2005; Senju, Kikuchi, Hasegawa, Tojo, & Osanai, 2008; von Grünau & Anston, 1995; but see Cooper, Law, & Langton, 2013).

The SITCE suggests that attention is more readily guided toward direct gaze than averted gaze targets, but the mechanisms underpinning such guidance remain unclear. At one extreme, it may reflect explicitly social processing in search mechanisms that guide attention across multiple-gaze displays (e.g., Baron-Cohen, 1994; Baron-Cohen, 2005 (the Eye Direction Detector model); Becchio, Bertone, & Castiello, 2008; Mareschal, Calder, & Clifford, 2013; Mareschal, Otsuka, & Clifford, 2014; Pantelis & Kennedy, 2016); that is, by processes that explicitly code eye gaze *as gaze*. However, two simpler possibilities exist. First, it may simply be that direct gaze eyes are a physically more salient stimulus than direct – the human eye’s morphology may give rise to a stronger luminance-contrast signal for direct gaze than averted, guiding attention without the observer needing to code gaze at all (e.g., Conty, George, & Hietanen, 2016; Lyyra, Astikainen, & Hietanen, 2018; Yokoyama, Ishibashi, Hongah, & Kita, 2011). Second, given direct gaze’s presumed importance, human vision may incorporate a perceptual *template* (bottom-up or top-down) that detects direct gaze in retinal input and guides attention toward it – such a template might be unconscious (e.g., Madipakkam, Rothkirch, Guggenmos, Heinz, & Sterzer, 2015) or innately specified (e.g., Farroni, Csibra, Simion, & Johnson, 2002; Senju & Johnson, 2009) and need only specify direct gaze’s visual features (a combination of sclera and circular iris features).

As these two alternative possibilities attest, human vision could prioritise direct gaze without coding gaze *as gaze.* Because direct gaze eyes form a very particular stimulus set with perceptual features that are distinct from averted gaze, it is difficult to disentangle which of the above mechanisms is operating in multiple-face arrays. Here, to sidestep these difficulties of interpretation, we instead examined search for eyes gazing toward *a prominent object* versus gazing away. Crucially, the eyes and faces in these two conditions were, across trials, identical (though of course different in each display), differing only in terms of the spatial relationship between the eyes’ gaze-direction and the object’s location. Accordingly, any enhanced search for eyes gazing *toward* an object (relative to eyes gazing away) could not reflect local physical characteristics or perceptual templates. These new conditions provide a previously unexploited opportunity to study sociocognitive processes involved in attentional guidance during search, helping to circumvent the difficulties of interpretation for other social stimuli (highlighted by Vestner, Gray, & Cook, 2020).

## Experiment 1: Influence of gazing *at* versus *away* when task-irrelevant

### Methods

The basic task in Experiment 1 was to detect the presence of target eyes in search displays, on the basis that they gazed in the opposite direction to the other eyes. Our manipulation of interest, whether the target eyes gazed toward the prominent object (hereafter, ‘Congruent Gaze’) or away (‘Incongruent Gaze’), was irrelevant for the observer. One concern with this design was that observers might opt to use a ‘singleton detection mode’ (e.g., Bacon & Egeth, 1994), attempting to detect physical discontinuities in the display rather than processing gaze. Accordingly, prior to 75% of search displays, a cue signalled to the observer which gaze direction (left or right) the target would have, to encourage them to search on the basis of gaze. The remaining 25% of trials comprised no cue. The Cued trials were designed to be more frequent to maximise the likelihood that observers would use the cue when it arose.

### Observers and sample size

For Experiment 1, we estimated that 24 observers should suffice to detect medium-large effects (Cohen’s f = 0.33/Cohen’s d= 0.65) of interest in a repeated-measures ANOVA with one group and two measurements (G*Power 3.0 software; Faul, Erdfelder, Lang, & Buchner, 2007). Twenty-four university students (*m* = 18, *f* = 6, ages 18–35 years) were recruited from posters and an online volunteer recruitment system and paid £7 for participating. The study was approved by the University of Cambridge Psychology Research Ethics Committee.

### Apparatus and stimuli

**Fig. 1:**
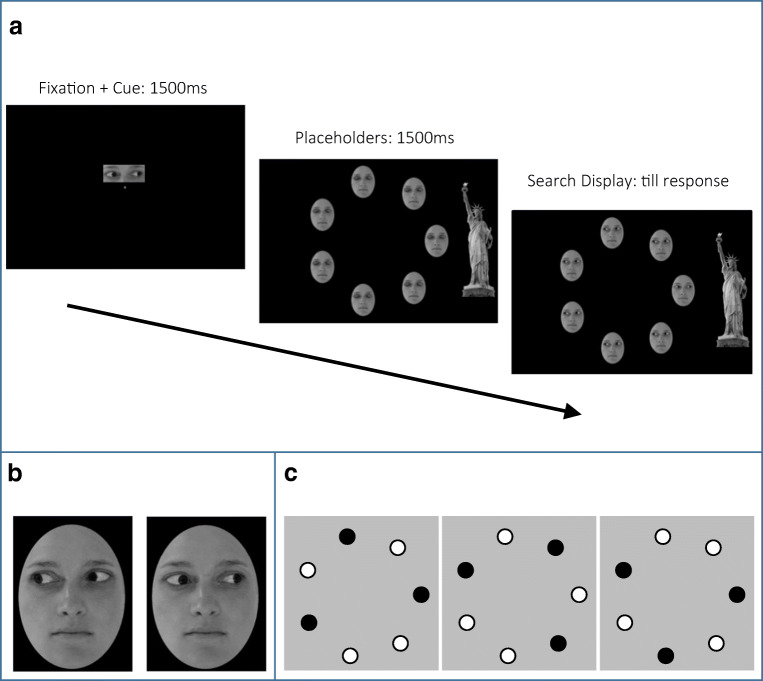
**a** Schematised sequence of displays in a typical Cued Trial from Experiment 1 (Set Size 7, Congruent Gaze Target), **b** left- and right-averted gaze faces from search displays, **c** three different arrangements used in Set Size 3 displays (black circles indicate positions of face stimuli, white circles positions left blank, with no face)

Observers sat 70 cm from a 24-in. Dell monitor (model number SE2416H, screen resolution 1,920 × 1,080), and made responses using a standard USB keyboard. Stimuli were presented using E-Prime 2.0 software (Psychology Software Tools Inc., 2013). Search displays were arrays of oval-cropped, forward-facing faces looking either to the left or to the right of the observer. To create the images, eyes from left-/right-gazing images were pasted onto the same closed-eyes image, so that only the eyes differed between the two (Fig. 1b). A Statue of Liberty image (hereafter ‘SoL’, downloaded from an open-source image database) was converted to grayscale and formed the prominent object toward which, or away from which, eyes might gaze in a display.

**Fig. 2:**
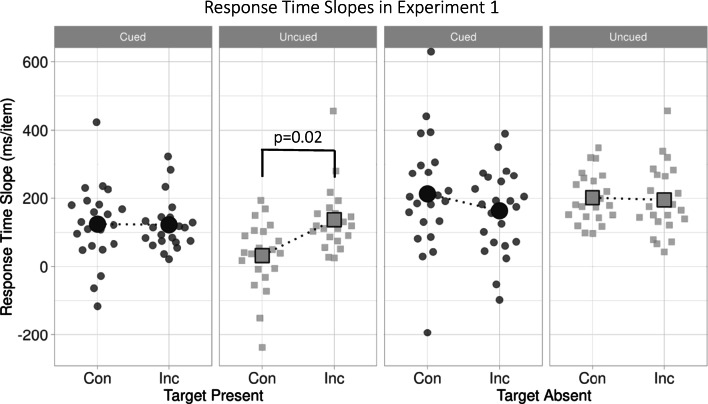
Response-time slopes for Congruent (‘Con’) versus Incongruent (‘Inc’) Gaze target trials in Cued conditions (dark grey circles) and Uncued conditions (light grey squares) of Experiment 1. Each point is an individual observer’s slope for each condition

Each individual face (279 × 370 pixels) subtended a visual angle of approximately 6° × 8° retinal angle. Faces were arranged in seven fixed positions spaced evenly on an imaginary circle (of 258-mm diameter) centred on fixation. In Set Size 7 displays, there was one face at each position. Set Size 3 displays were equally and unpredictably assigned to three configurations to prevent clustering in any one region of the display (see Fig. 2d and legend). Within each Set Size 3 configuration, and at Set Size 7, the target appeared equiprobably and unpredictably at any of the item locations. The Cue, on Cued Trials (see leftmost panel, Fig. 1a), was an enlarged snapshot of the eye region from the left or right gazing faces (105 × 96 pixels), subtending an angle of approximately 2° square. An SoL image (329 × 839 pixels), subtending a visual angle of approximately 7° × 18°, was placed either to the left or to the right of each search display (Fig. 1a, second and third panels).

### Procedure

Figure 1a schematises a typical display sequence in trials from Experiment 1. Each trial presented a fixation cross (1,500 ms), then a ‘placeholder’ display of three or seven faces with eyes closed (250 ms) followed by a search display of the same faces with eyes opened, lending a naturalistic impression of eyes opening. The presence of placeholder stimuli served to minimise distracting effects of non-eye facial features by cueing observers to the eye regions of the faces. At the same time that the placeholder stimuli appeared, the SoL image also appeared on screen, either to the right or left of the faces. A tall object was chosen such that faces at any position on the screen might conceivably be looking at the object. Observers were instructed to find the odd-one-out, unique target gaze in the display (present, unpredictably, on half of trials) when the eyes opened and make one of two keyboard responses as quickly as possible to indicate whether a target was present or not. Within each block, every combination of Set Size (Three, Seven), Target Presence (Present, Absent), and Target Gaze (Congruent, Incongruent) were equally represented, 75% of trials comprising a cue. Targets were either left-gazing eyes among right-gazing distracters or vice versa.

As shown in Fig. 1a, on 75% of trials, a pictorial target cue signalling the gaze of the unique target face in the subsequent search display informed observers as to which direction (left or right) the unique target eyes would gaze (no cue on remaining 25%). Orthogonally to this factor, Gaze Congruence (whether the target, if present, gazed toward the SoL or not) was manipulated: whether the target eyes gazed toward the SoL (Congruent Target Gaze) and the distracters gazed away) or vice versa (Incongruent Target Gaze). This congruency in Experiment 1 was entirely task-irrelevant – observers were only instructed to detect the unique gaze in each display. The search task began with a practice block (12 cued trials, three trials of each unique combination, and four uncued trials, one trial of each unique combination). Observers received feedback on their responses (“Correct!” = correct response, “------” = incorrect response). The main experimental trials followed, with no feedback, and were presented as five blocks of 64 trials, with each block having the same ratio of Cued to Uncued trials. Each block was followed by a 10-s break.

We predicted that search would be more efficient for congruent gaze eyes (looking toward the prominent SoL image) than incongruent gaze ones (looking away from the SoL). This prediction should be expressed as smaller search ‘slopes’ (RT increments from Set Size 3 to 7) for Target-Present trials only, as in Target-Absent trials all faces would have the same gaze so would compete equally for attention. Target-Absent trials were also analysed to confirm that any effect in Target-Present trials was not negated by an opposing effect in Target-Absent trials, which would point to a difference of response bias, rather than search efficiency. We made no strong predictions with respect to whether the effects would be stronger in Cued than Uncued Trials – this manipulation served only to enhance the potential to observe a gaze-congruence effect.

### Results and discussion

For each observer, response-time (RT) data for accurate responses were trimmed to exclude any RTs ± 3 standard deviations (SDs) for Cued and Uncued trials separately, for each combination of Target Presence, Congruence, and Set Size (our standard, data treatment as described in Ramamoorthy et al., 2019). One observer’s data were excluded as their RTs exceeded ± 3 SD from sample mean; another’s data were lost due to a technical error during acquisition.

Figure 2 plots mean RT slopes for Congruent and Incongruent trials, separately for Target-Present (left two panels) and Target-Absent (right two panels) trials. While search slopes (increases in RT from Set Size 3 to 7) appeared similar for Congruent and Incongruent trials in the Target-Absent condition, the same could not be said for the Target-Present condition. Instead, there appeared to be no effect of Congruence for Cued trials, but the predicted effect of Congruence for Uncued trials (shallower search slopes for Congruent than Incongruent trials).

For brevity, as our terms-of-interest all involved search efficiency, we simplified the analyses described here by calculating search slopes for each condition and observer (RT for Set Size 7 minus RT for Set Size 3), using this as the dependent variable for ANOVA.^1^ Note, this choice did not impact any of our conclusions; mean RTs and accuracy for each condition are detailed in Table 1, separately for each set size. The omnibus three-way ANOVA with factors of Cue (Cued, Uncued), Congruence (Congruent or Incongruent), and Target Presence (Present or Absent) yielded no main effect of Congruence ([*F* (1, 21) = 1.923, *p = .180*, ${\eta}_p^2$= .264]), but main effects of Target Presence ([*F* (1, 21) = 46.483, *p < .001*, ${\eta}_p^2$= .689) and Cue ([*F* (1, 21) = 7.549, *p = .012*, ${\eta}_p^2$= .084]). There was no two-way interaction between Congruence and Target Presence ([*F* (1, 21) = 1.991, *p = .173*, ${\eta}_p^2$= .087]), and only marginal Cue × Congruence and Cue × Target Presence interactions ([*F* (1, 21) = 3.442, *p = .078*, ${\eta}_p^2$= .141], [*F* (1, 21) = 4.451, *p = .047*, ${\eta}_p^2$= .175], respectively). Crucially, there was a three-way interaction between Congruence, Target Presence and Cue ([*F* (1, 21) = 5.217, *p = .033*, ${\eta}_p^2$= .199]).

**Table 1:** Mean response times (RTs) and accuracy (Acc) (*SD in brackets*) for (**A**) Target-Present and (**B**) Target-Absent conditions across Set Sizes 3 and 7 for all experiments

**A**	Target-Present							
	Cued				Uncued			
	Congruent		Incongruent		Congruent		Incongruent	
	Set Size 3	Set Size 7	Set Size 3	Set Size 7	Set Size 3	Set Size 7	Set Size 3	Set Size 7
Experiment 1								
*RT*	1,306.21 *(555.07)*	1,853.75 *(805.05)*	1,243.82 *(406.63)*	1,732.67 *(587.91)*	1,357.43 *(586.91)*	1,486.07 *(511.33)*	1,344.38 *(581.69)*	1,840.61 *(711.20)*
*Acc*	0.94 *(0.06)*	0.89 *(0.10)*	0.95 *(0.05)*	0.88 *(0.08)*	0.92 *(0.09)*	0.90 *(0.11)*	0.97 *(0.07)*	0.87 *(0.16)*
Experiment 2								
*RT*	978.30 *(181.61)*	*1,358.23* *(229.57)*	*1,105.08* *(227.98)*	1,472.02 *(219.83)*	1,185.08 *(300.74)*	1,553.21 *(387.32)*	1,296.11 *(357.73)*	1,783.34 *(443.02)*
*Acc*	0.95 *(0.04)*	0.94 *(0.06)*	0.96 *(0.04)*	0.94 *(0.04)*	0.97 *(0.03)*	0.92 *(0.09)*	0.96 *(0.04)*	0.94 *(0.05)*
**B**	Target-Absent							
	Congruent		Incongruent		Congruent		Incongruent	
	Set Size 3	Set Size 7	Set Size 3	Set Size 7	Set Size 3	Set Size 7	Set Size 3	Set Size 7
Experiment 1								
*RT*	1,324.98 *(480.52)*	2,125.54 *(651.59)*	1,305.90 *(489.21)*	2,074.64 *(554.04)*	1,330.46 *(479.91)*	2,151.57 *(611.21)*	1,386.97 *(571.38)*	2,207.76 *(766.81)*
*Acc*	0.97 *(0.04)*	0.95 *(0.11)*	0.97 *(0.04)*	0.94 *(0.11)*	0.95 *(0.11)*	0.96 *(0.12)*	0.94 *(0.12)*	0.95 *(0.07)*
Experiment 2								
*RT*	1,097.52 *(162.34)*	1,885.33 *(370.66)*	1,171.85 *(187.06)*	2,050.16 *(446.60)*	1,224.45 *(285.90)*	2,123.49 *(67181)*	1,309.97 *(343.36)*	2,316.90 *(778.21)*
*Acc*	0.99 *(0.02)*	0.99 *(0.01)*	0.98 *(0.03)*	0.98 *(0.04)*	0.98 *(0.02)*	0.99 *(0.02)*	0.98 *(0.02)*	0.99 *(0.02)*

To investigate the source of this interaction, we first conducted independent, two-way ANOVAs for Target-Present and Target-Absent trials: an attentional bias toward targets over distracters could only operate in the former case. As expected on this view, for Target-Present trials, a two-way ANOVA revealed a marginal effect of Cue [*F* (1, 21) = 4.088, *p = .056*, ${\eta}_p^2$= .163], a main effect of Congruence [*F* (1, 21) = 8.299, *p = .009*, ${\eta}_p^2$= .395], and, crucially, an interaction of the two factors [*F* (1, 21) = 7.116, *p=.014*, ${\eta}_p^2$= .253]. This interaction reflected shallower search slopes (more efficient search) for Congruent than Incongruent Gaze targets in Uncued trials (t(21)= -2.518, *p= .020*) that were reduced or absent for Cued trials (t(21)= 1.051, *p= .305*). That is, the effect of Congruence was as predicted in Target-Present trials overall, but not with respect to its small size or absence in Cued, Target-Present Trials. Corresponding analyses for Target-Absent trials revealed no main effect or interaction (max *F* = 0.4, *n.s.*). This was consistent with the effect in Target-Present trials reflecting biased attentional guidance toward Congruent Gaze targets when they were present, rather than faster serial rejection of (congruent or incongruent) distracters – the latter would be typically associated with larger absolute effects of Congruence on RTs in Target-Absent trials than Target-Present ones.

We also investigated the three-way interaction by splitting the analysis into Cued and Uncued trials. This alternative analysis was perhaps less powerful, given the greater variability of Target-Absent search slopes, but perhaps more sensitive to effects common to both Target-Present and -Absent trials. The two-way ANOVA for Cued trials revealed only a main effect of Target Presence [*F* (1, 21) = 8.817, *p = .007*, ${\eta}_p^2$= .296], and no main effect of Congruence [*F* (1, 21) = 1.652, *p = .213*, ${\eta}_p^2$= .073], or interaction of the two [*F* (1, 21) = 0.138, *p = .714*, ${\eta}_p^2$= .007]. For Uncued trials, there was a significant main effect of Target Presence [*F* (1, 21) = 51.813, *p < .001*, ${\eta}_p^2$= .712], only a marginal main effect of Congruence [*F* (1, 21) = 2.972, *p = .099*, ${\eta}_p^2$= .124], and a marginal interaction of the two factors [*F* (1, 21) = 3.480, *p = .076*, ${\eta}_p^2$= .142]. While these findings were broadly consistent with the clear patterns in the primary analysis, the reliability of congruence effects, only observed here in Target-Present, Uncued trials, required confirmation in a further experiment.

## Experiment 2: Influence of task-relevant gaze congruence

### Method

The aim here was to more clearly examine the congruence effects observed in Experiment 1. Accordingly, Experiment 2 largely replicated the conditions of Experiment 1, but with two important exceptions. First, each observer now only experienced Cued or Uncued trials, yielding a completely independent assessment of Congruence effects for each Cue condition individually (see below, for details). Second, to maximise the opportunity to see Target Gaze Congruence effects (target gazing toward the SoL, vs. gazing away), this feature was now *task-relevant* : that is, the side on which the SoL was presented was 100% informative as to which side the target would gaze in any block of trials (half of the blocks of trials comprised only Congruent Gaze targets, the remainder, only Incongruent Gaze ones). Based on results from Experiment 1, our single important prediction was an effect of congruence for the Uncued Condition. We now made no strong predictions for the Cued Condition, which served only to clarify whether congruence effects could also be observed with a prior cue.

### Observers and sample size

For the two conditions of Experiment 2, the same calculations as for Experiment 1 were applied (24 observers per study). Note these conditions were run consecutively (not randomly assigned in advance). Accordingly, each condition is technically a separate experiment. Here, for brevity and clarity, they are described together to parallel the description of Experiment 1. The total sample (48 observers, *m* = 29, *f* = 19) was estimated to be sufficient for cross-study within-between interactions and between-observers main effects (Cohen’s f = 0.33, two groups, four measures, power = 0.8).

### Apparatus, stimuli and procedure

Cued and Uncued conditions replicated those of Experiment 1, with the following exceptions. First, trials were now divided into two blocks of 120 trials each, with each block of only one Congruence type (run order for Congruent and Incongruent blocks counterbalanced across observers). The side on which the SoL appeared (only 250 ms prior to the search display, along with placeholder faces) provided 100% valid cueing as to where the unique target gaze would be directed, thus making Target Gaze Congruence task-relevant. Second, while the Cued Condition supplemented this information with a 100% valid prior cue as to the direction of target gaze (left or right, as in Cued trials of Experiment 1), the Uncued Condition did not (as in Experiment 1).

### Results and discussion

**Fig. 3:**
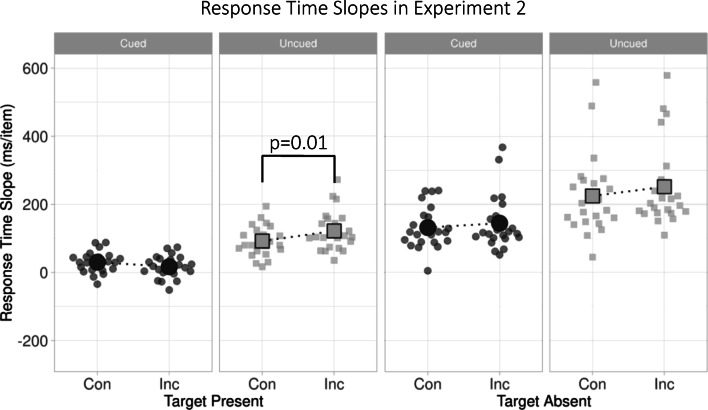
Response-time slopes for Congruent versus Incongruent gaze target trials in Cued conditions (dark grey circles) and uncued Conditions (light grey squares) of Experiment 2. Same format as Fig. 2

One observer had to be removed from each of the Conditions, as even post trim their RTs exceeded ± 3 SD from sample mean. Figure 3 plots RTs for Cued and Uncued conditions in the same format as Fig. 2. As for Experiment 1, analyses were conducted on RT search slopes.^2^ An omnibus three-way ANOVA (factors of Congruence and Target Presence as for Experiment 1, Cue now between-observers) revealed standard effects of Target Presence [*F* (1, 44) = 111.669, *p < .001*, ${\eta}_p^2$= .717], Congruence [*F* (1, 44) = 12.087, *p < .001*, ${\eta}_p^2$= .215] and a marginal Congruence by Cue interaction [*F* (1, 44) = 2.914, *p = .095*, ${\eta}_p^2$= .062] but no other main effects or interactions (max *F* = 1.2 , *n.s.*). These findings provided strong evidence that search for Congruent targets is more efficient than for Incongruent ones. Evidence for any modulation of this effect by the presence of a Cue was relatively weak.

To parallel our analyses for Experiment 1, we conducted a two-way mixed ANOVA on RT slopes from Target-Present trials only. This yielded a marginal effect of Congruence [*F* (1, 22) = 3.538, *p = .067*, ${\eta}_p^2$= .074], no main effect of Cue [*F* (1, 22) = 1.682, *p = .201*, ${\eta}_p^2$= .037], but an interaction between the two factors [*F* (1, 22) = 5.484, *p = .024*, ${\eta}_p^2$= .111]. This, as in Experiment 1, reflected more efficient search for Congruent than Incongruent targets for Uncued [t(22)= -2.833, *p= .010*] but not Cued trials [t(22)= .346, *p= .733*]. However, in contrast to Experiment 1, the corresponding ANOVA on Target-Absent trials yielded a main effect of Congruence [*F* (1, 22) = 9.419, *p = .004*, ${\eta}_p^2$= .176], but no main effect of Cue [*F* (1, 22) = 1.171, *p = .285*, ${\eta}_p^2$= .026], and a marginal interaction between the two factors [*F* (1, 22) = .072, *p = .789*, ${\eta}_p^2$= .002]. There was now strong evidence of Congruence effects across Target-Present and Target-Absent trials. The influence of Cue on this effect was now less clear.

To assess whether Uncued Trials showed effects of Congruence when considered in isolation, an individual ANOVA on the Uncued Condition Slopes was conducted. This found main effects of Congruence [*F* (1, 22) = 10.609, *p = .004*, ${\eta}_p^2$= .325] and Target Presence [*F* (1, 22) = 46.104, *p < .001*, ${\eta}_p^2$= .677], with no evidence of an interaction between the two [*F* (1, 22) = .030, *p = .863*, ${\eta}_p^2$= .001]: more efficient search for Congruent than Incongruent targets, largely *irrespective* of Target Presence. Corresponding analyses of Cued trials yielded no main effect of Congruence [*F* (1, 22) = 2.134, *p = .158*, ${\eta}_p^2$= .037], a main effect of Target Presence [*F* (1, 22) = 1.682, *p < .001*, ${\eta}_p^2$= .088], and a marginal interaction [*F* (1, 22) = 3.658, *p = .069*, ${\eta}_p^2$= .143]. Search was marginally more efficient in Congruent than Incongruent trials, but only in the Target-Absent condition.

Experiment 2 therefore provided strong confirmation of our claim here: that search for congruent gaze (eyes looking *at* a prominent object) is more efficient than incongruent gaze (eyes looking elsewhere). Again, contrary to our concern prior to Experiment 1 that Congruence effects might only arise in Cued trials, this effect was again most clearly evident in Uncued trials. However, while Congruence effects were unclear in Cued Trials, overall they showed the same numerical trend as for Uncued trials (*p = 0.158*). It therefore seems likely that the effect of adding a cue to Cued trials is not to eliminate the congruence effect, but to render it more difficult to detect; the cue likely biasing attention toward the target’s gaze direction, thus obscuring any subtler effects of gaze-congruence.

## General discussion

The current experiments were motivated by previous work on the ‘Stare in the Crowd’ effect (SITCE): more efficient search for *direct* gaze (towards the observer) than for averted gaze. That effect does not provide clear evidence of social-cognition influences on search as it might, instead, reflect intrinsic salience of direct-gazing eyes or a specific perceptual-template tuned to direct gaze. Here, we found that search for congruent gaze (toward *an object*) is more efficient than for incongruent gaze (away from it). In this novel comparison, face and eye stimuli in these two conditions were identical (across trials), precluding explanation in terms of local templates or intrinsic salience. Congruent and Incongruent gaze differed only in their *spatial relationship* to a prominent object, so the observed advantage for Congruent Gaze must have reflected this.

One possibility is that the congruent gaze advantage reflects processes in search that explicitly code gaze *as gaze,* perhaps the same processes that determine conscious perception of gaze when observers give unhurried attentional scrutiny to one face. In such a case, the congruent gaze advantage might be shaped by the high-level, theory-of-mind-related processes identified in non-search tasks (Hamilton, 2016; Teufel, Fletcher, & Davis, 2010). However, such rich processing is not typical of the types of representations found to guide visual search (Wolfe & Horowitz, 2017). Neither is it required to explain our results. At a minimum, the process identified here need only code eye-gaze images as ‘pointing’ left or right, plus the spatial relationship of eye-gaze and object. Accordingly, therefore, we speculate that the social or ‘protosocial’ processes underpinning the gaze-congruence effect will be specific to social stimuli yet lack the theory-of-mind-related influences established for individually attended faces.

Another high-profile recent claim that social processes can influence search has been based on detection of social dyads: silhouette figures facing each other or away (Vestner et al., 2020). That study suggested that more efficient search for human figures facing toward than away from each other in those displays may be ascribed to alternative explanations in terms of stimulus confounds arising because the two interacting figures in those experiments are side by side. Here, by contrast, the gaze-congruence effect reflects a relationship between target eyes and an object that typically were distant from one another – those same concerns cannot apply.

Finally, a challenging question facing any study of attention-guidance in search is whether shallower search-slopes are better explained by faster serial selection and rejection of items in a display or by noisy, inefficient, yet parallel guidance. As Wolfe and Horowitz (2017) note, previous findings with regard to potential higher-level influences in search (in that case, for facial expressions) are often amenable to explanation in terms of self-terminating serial search models *with no parallel guidance component*. These latter models would predict larger absolute effects on target-absent trials than target-present trials. Here, in contrast, our gaze-congruence effect was only clearly evident in target-present trials: inconsistent with that model and exactly as predicted by attention-guidance views. While parallel and serial models can, in principle, both be extended and contorted to accommodate any new finding, our results are more readily explained as gaze-congruence influences on attention guidance. On this basis, the current results provide much clearer evidence for social or proto-social guidance of attention.

One unresolved issue in the current experiments is whether a prior cue (in Cued trials) suppresses the gaze congruence effect or merely obscures it. In Experiment 2, there was a strong overall effect of congruence, but still not clearly evident for Cued trials considered in isolation. We speculate that the cue in Cued trials likely induces an attentional bias toward the target’s eye gaze and that this obscures expression of gaze congruence effects. Future research should unpick the particular contributions of these two types of effects on search. However, this does not impact our central claim here, regarding the existence of congruence effects.

In summary, these findings provide the clearest evidence to date that social processes influence visual search for gaze. Our findings cannot be accounted for by local salience, perceptual-templates or other stimulus confounds. Whether the gaze coding that drives search efficiency is as sophisticated as that which attends our perception and cognition of single, attended faces remains to be seen. However, gaze-congruence effects demand explanation in terms of spatial-relationships between gazing eyes and objects in search: a much richer process than has previously been demonstrated in search for gaze.

## Data Availability

The data and materials for the experiments reported here will be made available upon request.

## Footnotes

1 Our analyses focus on RT slopes as the primary dependent variable. Accuracy scores (see Table 1) were generally high (*M* = 0.94, *SD* = 0.10) and an identical omnibus ANOVA to that for RTs revealed no effects involving Congruence (max *F* = 3.259, *n.s*.) that might threaten our interpretation of the RT data.

2 Accuracy was again high (Cued: *M*= 0.97, *SD* = 0.03, Uncued: *M*= 0.97, *SD* = 0.03); an identical omnibus ANOVA to that run on RTs revealed no effects involving Congruence (max *F* = 3.065, *n.s.*) that might threaten our interpretation of the RT data
